# Norovirus infections in young children in Lusaka Province, Zambia: clinical characteristics and molecular epidemiology

**DOI:** 10.1186/s12879-017-2206-2

**Published:** 2017-01-23

**Authors:** Leigh M. Howard, Innocent Mwape, Mpanji Siwingwa, Michelo Simuyandi, M. Brad Guffey, Jeffrey S. A. Stringer, Benjamin H. Chi, Kathryn M. Edwards, Roma Chilengi

**Affiliations:** 1 Department of Pediatrics, Division of Pediatric Infectious Diseases, Vanderbilt University Medical Center, D-7228 MCN; 1161 21st Ave S, Nashville, TN 37232 USA; 2 Centre for Infectious Disease Research in Zambia (CIDRZ), P.O. Box 34681, Lusaka, 10101 Zambia; 3 Division of Global Women’s Health, University of North Carolina (UNC) – Chapel Hill, 130 Mason Farm Rd., 2nd Floor, Campus Box #7030, 27599-7030 Chapel Hill, NC USA

**Keywords:** Norovirus, Young children, Zambia, Diarrhea, Gastroenteritis

## Abstract

**Background:**

The burden, clinical features, and molecular epidemiology of norovirus infection in young children in southern Africa are not well defined.

**Methods:**

Using data from a health facility-based surveillance study of children <5 years in Lusaka Province, Zambia presenting with diarrhea, we assessed the burden of norovirus infection. A convenience sample of 454 stool specimens was tested for norovirus using reverse-transcriptase polymerase chain reaction (RT-PCR). RT-PCR positive samples underwent additional nucleotide sequencing for genogroup and genotype identification. Clinical features and severity of diarrheal illnesses were compared between norovirus-positive and -negative subjects using Chi-squared and t-tests.

**Results:**

Norovirus was detected in 52/454 (11.5%) specimens tested. Abdominal pain, fever, and vomiting were the most common presenting features in norovirus-associated illnesses. However, there were no significant differences in the clinical features of norovirus-positive compared to norovirus-negative illnesses. Of 43 isolates that were available for sequencing, 31 (72.1%) were genogroup II (GII) and 12 (27.9%) were genogroup I (GI). The distribution of genotypes was diverse.

**Conclusions:**

Noroviruses were detected in approximately 10% of young children with diarrhea in the Lusaka Province of Zambia, with GII representing the majority of infections. These findings support the role of norovirus in symptomatic diarrhea disease in Africa. Further studies are needed to confirm these observations and to evaluate prevention strategies.

**Electronic supplementary material:**

The online version of this article (doi:10.1186/s12879-017-2206-2) contains supplementary material, which is available to authorized users.

## Background

Norovirus (NoV) is a common cause of acute gastroenteritis (AGE) worldwide. NoVs are members of the *Caliciviridae* family and are divided into six genogroups, of which genogroups I and II (GI and GII) are responsible for the majority of human disease [1, 2]. Each genogroup can be further distinguished into genotypes, with GI and GII comprised of at least 9 and 22 genotypes, respectively [2]. Most human NoV infections are caused by GII strains, and a single genotype, GII.4, is responsible for approximately 55–85% of clinical cases worldwide [2].

NoV is the most common cause of acute gastroenteritis in outbreak settings [3]. However, NoV is also a leading cause of AGE among children in the community. Since the introduction of rotavirus vaccines, NoV has replaced rotavirus as the leading cause of medically attended AGE in children < 5 years of age in the United States [4, 5]. However, the burden of norovirus-associated diarrheal infections in the pre- and post-rotavirus vaccination era has not been fully characterized in other regions, including southern Africa. We aimed to evaluate the prevalence, clinical features, and molecular epidemiology of NoV infections in children < 5 years of age in Zambia, a country that implemented infant rotavirus vaccination in 2013, using data from a health facility-based surveillance study.

## Methods

### Study setting

The study was conducted in six health facilities throughout the Lusaka Province of Zambia. Three facilities were in Lusaka District, the most densely populated urban district in Zambia and three additional facilities were located in each of the three remaining districts, Kafue, Chongwe and Luangwa, which contain a lower density, more rural population. In January 2012 the Zambian Government, in partnership with the Centre for Infectious Disease Research in Zambia (CIDRZ) and Absolute Return for Kids (Ark, a United Kingdom-based children’s charity), initiated a 2-year, pilot introduction of the Rotarix® live, oral rotavirus vaccine in public health facilities in Lusaka Province [6, 7]. Following this successful pilot, the vaccine was nationally implemented into the Expanded Programme on Immunisations (EPI) in November 2013. Facilities that met specific criteria were purposefully selected as study sites, including (i) sufficient numbers of patients under 5 years, (ii) space to support study activities, (iii) and participation in the pilot vaccine roll-out.

### Study design

From July 2012 to October 2013, active health facility-based surveillance was conducted among the six sentinel health facilities to identify and enroll children less than 5 years of age with diarrheal illnesses. Children were considered eligible if they met the following criteria: age 0–59 months; admitted to the inpatient department (IPD) or under care in the outpatient department (OPD) at the time of screening; and verbal confirmation by the caregiver that the child passed three or more abnormally loose stools in the past 24 h. Eligibility criteria also including having signs of potentially severe diarrhea by confirmation of at least one of the following symptoms assessed through physical examination by the study nurse or verbal confirmation by the caregiver: sunken eyes, loss of normal skin turgor, intravenous rehydration prescribed/administered, blood in stool, and hospitalization for diarrhea or dysentery. Inclusion criteria also included presence of a caregiver who could provide written informed consent and who was willing to have study procedures carried out on the eligible child. Subjects who had previously participated in the study within the last 30 days were excluded.

After providing written informed consent, caregivers of participants were interviewed at enrollment to collect detailed information regarding individual and household demographics, symptoms associated with the child’s present illness, and the child’s health background. The child’s vaccination history and growth trajectory were also recorded from the child health record, when available, or by verbal report by caregiver when not. If the child was admitted to the health facility, time-to-discharge and other features of illness were collected. A home visit was also conducted 30 days after enrollment or discharge from the facility to determine the child’s vital status.

### Stool sample collection and handling

Study staff asked the child’s caregiver to be notified when the child indicated a need to use the toilet or after the child had produced stool in the diaper. Approximately 10–15 mL of bulk stool was collected from each child at enrollment. Stool specimens were collected in sterile specimen containers, refrigerated immediately after collection, and transported from the study sites to the CIDRZ laboratory in Lusaka.

### Laboratory procedures

All stool samples collected were initially screened for rotavirus antigen by enzyme immunoassay (EIA) using a monoclonal antibody solid phase sandwich as part of a larger assessment of rotavirus vaccine effectiveness [8]. The Meridian Rotaclone EIA kit for the detection of rotavirus antigen in fecal samples (CAT No. 696004) was used for the analysis.

### Norovirus RNA isolation and detection

A convenience sample of available stool specimens was processed to undergo norovirus testing by reverse-transcriptase polymerase chain reaction (RT-PCR). RNA was extracted from 140 μL of fecal suspension using the QIAampViral RNA Mini Kit, (Qiagen, Hieden, Germany). Purified RNA aliquots were stored at −80 C. One step RT-PCR was performed on the stored samples using the JV12i/JV13y primers targeting the ORF1 region, which encodes the RNA-dependent RNA-polymerase gene [9]. The final volume for each reaction was 25 μL. Each reaction contained 5.0 μL of 5x one step RT-PCR buffer; 1.0 μL of 40 μM dNTPs; 0.5 μL of each primer for (20 μM each); 1 μL of One step RT-PCR Enzyme mix (5 U/μL), 5 μL of sample RNA, and 11 μL of nuclease-free water. The amplification conditions were set as follows: Reverse transcription 42 °C for 30 min, followed by 94 °C for 5 min, then 40 cycles of 94 °C for 30 s, 50 °C for 30 s, 72 °C for 30 s, and an extension step at 72 °C for 10 min. All RT-PCR products were analyzed by 1.5% agarose gel electrophoresis.

### DNA sequencing and genotyping

All positive RT-PCR products were purified using GeneJET PCR Kit (Thermo Scientific) and sequenced with BigDye Terminator v3.1 cycle sequencing kit using an ABI 3130 XL Genetic analyzer (Applied Biosystems, Foster City, CA). Nucleotide sequences were edited with Sequencer Version 5.0. The partial RNA-dependent RNA-polymerase genotypes of sequenced isolates were determined using the Norovirus Genotyping Tool Version 1.0 (National Institute for Public Health and the Environment; Bilthoven, The Netherlands). Phylogenetic relationships of the norovirus were analyzed by aligning sequences from the GenBank database using clustalw software. Phylogenetic trees of nucleotide sequences of the partial RNA-dependent RNA polymerase region of GI or GII isolates were constructed by the neighbor joining method [10] using MEGA version 5.0 software [11] and validated by 1000 bootstrap replicates [12].

### Human subjects protection

The study was approved by research ethics authorities at the Zambian Ministry of Health, the University of Zambia, the University of Alabama at Birmingham, and the University of North Carolina at Chapel Hill. Caregivers of participants provided written informed consent prior to initiation of study procedures.

### Statistical analysis

The distribution of genogroups among the norovirus detections sequenced and the seasonality of norovirus detections were described. To compare the clinical features of AGE among norovirus-positive versus norovirus-negative episodes of diarrheal illness, Chi-squared tests and t-tests were utilized for categorical or continuous outcome variables, respectively. Severity scores were calculated post-hoc for each diarrheal illness for which clinical data were available using the 20-point Modified Vesikari score [13–15] and compared among norovirus genogroup and according to viral detection.

## Results

### Norovirus prevalence and distribution of genogroups and genotypes

**Fig. 1:**
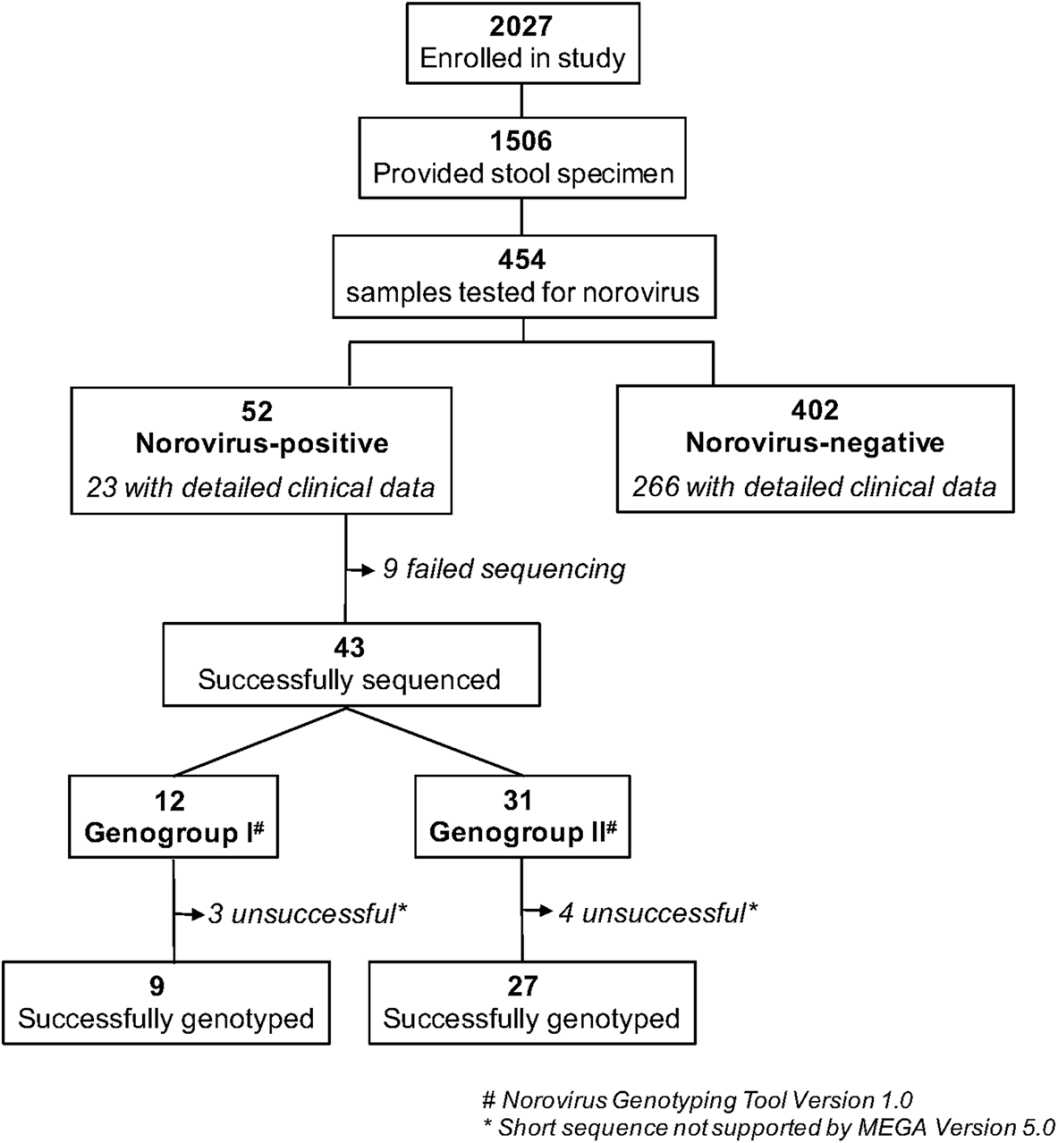
Consort diagram

A total of 1506 subjects were enrolled in the primary study and provided stool specimens. Rotavirus positivity among these was 41.2% (621/1506). From 1506 stool specimens collected during the study, a convenience sample of 454 (30%) specimens was selected for norovirus testing. Overall, 52/454 (11.5%) of these specimens were positive for norovirus by RT-PCR (Fig. 1). Of 52 norovirus-positive samples, 43 (82.7%) were successfully sequenced for genogroup characterization. The majority of these (31/43; 72.1%) were GII, while 12/43 (27.9%) were GI.

Genotype determination was not possible in 7/43 samples due to short sequences not supported by MEGA V.5. In the 36 strains genotyped, there were 9 GI samples and 27 GII samples. Of 9 GI strains successfully genotyped, strains clustered as follows: 6 GI.P7, 2 GI.P2, and 1 GI.P5 (Additional file 1: Figure S1A). Of 27 GII strains successfully genotyped, strains clustered as follows: 19 GII.P4, 4 GII.Pe and 4 GII.P2 (Additional file 1: Figure S1B).

### Clinical features of norovirus-positive compared to norovirus-negative diarrheal illnesses

**Fig. 2:**
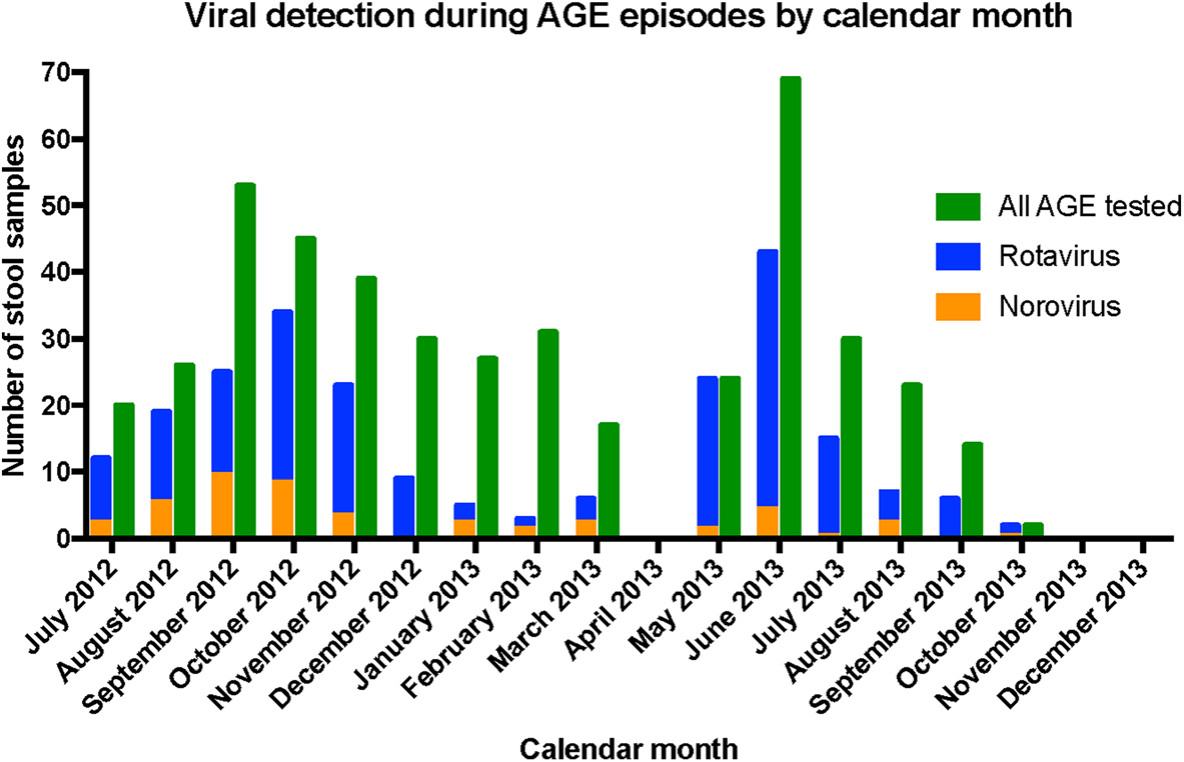
Calendar month of collection and norovirus detection among samples collected from young Zambian children with diarrheal illness

Detailed clinical and sociodemographic data were available for 289/454 (63.7%) samples, including 23 of the 52 (44.2%) in which norovirus was detected (Table 1). The mean duration of diarrhea was 2.9 days in these 23 norovirus-positive diarrheal illnesses; no subjects reported bloody diarrhea. Abdominal pain, fever, and vomiting were the most common features present during norovirus infections, and occurred in 15/23 (68.1%), 10/23 (45.4%), and 9/23 (42.9%) norovirus-positive episodes, respectively. Rotavirus was co-detected in 6/23 (26%) episodes of norovirus-positive diarrheal illness; two of these subjects had received one rotavirus vaccine dose, but none had received two rotavirus vaccine doses. NoV detections occurred throughout the year, with no discernible seasonal pattern (Fig. 2). Ten of 23 (43%) subjects with norovirus infection and clinical data available had received at least one rotavirus vaccine dose. In the subjects in whom all data were available to calculate a Modified Vesikari Score, most (16/19; 84.2%) illnesses were characterized as mild, and the remainder (3/19; 15.8%) were characterized as moderate. Three of 21 (14.3%) children with norovirus-positive diarrheal illness were admitted to the health care facility. None of these children were also rotavirus-positive; length of stay for each admitted child was 2 days.

**Table 1:** Sociodemographic and clinical features of norovirus-positive compared to norovirus-negative diarrheal illness episodes in young Zambian children

	Norovirus positive (*n* = 23)	Norovirus negative (*n* = 266)	*p*
Sociodemographic and background characteristics			
Sex			0.240
Female	14/22 (63.6)	127/257 (49.4)	
Male	8/22 (36.3)	130/257 (50.6)	
Age			0.797
< 1 month	0 (0.0)	3/266 (1.1)	
1–6 months	2/23 (8.7)	31/266 (11.7)	
6–12 months	10/23 (43.5)	106/266 (39.8)	
12–24 months	9/23 (39.1)	83/266 (31.2)	
> 24 months	2/23 (8.7)	43/266 (16.2)	
Number of siblings			0.867
0	8/21 (38.1)	69/210 (32.9)	
1	4/21 (19.1)	53/210 (25.2)	
2	4/21 (19.1)	48/210 (22.9)	
3	2/21 (9.5)	17/210 (8.1)	
≥ 4	3/21 (14.3)	23/210 (11.0)	
Household electricity	15/23 (65.2)	164/258 (63.6)	0.875
Household drinking water source			0.793
Communal tap	12/19 (63.1)	110/244 (45.1)	
Piped to yard	3/19 (15.8)	39/244 (16.0)	
Piped into dwelling	2/19 (10.5)	36/244 (14.8)	
Protected public well	1/19 (5.3)	21/244 (8.6)	
River/stream	0/19 (0.0)	14/244 (5.7)	
Other	1/19 (5.3)	24/244 (9.8)	
Household livestock	3/23 (13.0)	28/251 (11.2)	0.784
Toilet facility			**0.031**
Flush/pour flush to piped sewer	1/20 (5.0)	41/225 (18.2)	
Flush/pour flush to septic tank	3/20 (15.0)	42/225 (18.7)	
Flush/pour flush to pit latrine	3/20 (15.0)	3/225 (1.3)	
Improved ventilated pit latrine	1/20 (5.0)	16/225 (7.1)	
Pit latrine with slab	10/20 (50.0)	78/225 (34.7)	
Open pit latrine without slab	2/20 (10.0)	44/225 (19.6)	
Hanging toilet/hanging latrine	0/20 (0.0)	1/225 (0.4)	
Child ever diagnosed with HIV/AIDS	0/18 (0.0)	1/201 (0.5)	1.000
Number of rotavirus vaccines received			
0 doses or unknown	13/23 (56.5)	171/266 (64.3)	
At least 1 dose	10/23 (43.4)	95/266 (35.7)	0.458
2 doses	8/23 (34.8)	79/266 (30.0)	0.610
Clinical features at presentation			
Severity score by category			0.766
≤ 10 (mild)	16/19 (84.2)	156/183 (85.3)	
11–14 (moderate)	3/19 (15.8)	26/183 (14.2)	
≥ 15 (severe)	0/19 (0.0)	1/183 (0.6)	
Degree of dehydration on admission			0.319
None	2/23 (8.7)	57/259 (22.0)	
Mild	7/23 (30.4)	55/259 (21.2)	
Moderate	14/23 (60.9)	136/259 (52.5)	
Severe	0/23 (0.0)	11/259 (4.3)	
Duration of diarrhea, days			0.505
1–4	21/22 (95.5)	203/221 (91.9)	
5	1/22 (4.5)	6/221 (2.7)	
≥ 6	0/22 (0.0)	12/221 (5.4)	
Max stools/day			0.444
1–3	14/22 (63.6)	112/217 (51.6)	
4–5	8/22 (36.4)	90/217 (41.5)	
≥ 6	0/22 (0.0)	15/217 (6.9)	
Blood in stool	0/22 (0.0)	8/216 (3.7)	1.000
Vomiting	9/21 (42.9)	83/221 (37.1)	0.603
Duration of vomiting, days			0.593
0	12/22 (54.6)	139/221 (62.9)	
1	1/22 (4.6)	9/221 (4.1)	
2	5/22 (22.7)	49/221 (22.2)	
≥ 3	4/22 (18.2)	24/221 (10.9)	
Maximum vomiting episodes/day			0.502
0	12/22 (54.6)	139/218 (63.8)	
1	0/22 (0.0)	5/218 (2.3)	
2–4	10/22 (45.5)	65/218 (29.8)	
≥ 5	0/22 (0.0)	9/218 (4.1)	
Abdominal pain	15/22 (68.2)	137/205 (66.8)	0.898
Caregiver-reported fever	10/22 (45.5)	97/217 (44.7)	0.946
Maximal recorded temperature, °C			0.877
< 37.0	15/22 (68.2)	172/245 (70.2)	
37.1–38.4	7/22 (31.8)	60/245 (24.5)	
38.5–38.9	0/22 (0.0)	6/245 (2.5)	
≥ 39.0	0/22 (0.0)	7/245 (2.9)	
Duration of fever	2.4 (1.2)	2.6 (1.3)	0.605
Decreased energy/malaise	13/21 (61.9)	147/217 (67.7)	0.586
Excessive crying	14/22 (63.6)	96/218 (44.0)	0.079
Admitted to hospital	3/21 (14.3)	32/223 (14.4)	1.000
Rotavirus positive	6/23 (26.1)	104/263 (39.5)	0.203
30 day follow-up			
Status of child			1.000
Fully recovered	10/10 (100.0)	120/133 (90.20)	
Partially recovered	0/10 (0.0)	6/133 (4.5)	
Worsening	0/10 (0.0)	1/133 (0.8)	
Recovered but became sick again	0/10 (0.0)	6/133 (4.5)	

Bold text indicates statistical significance (*p* < 0.05)

The types of household toilet facilities available varied significantly between those with norovirus-positive compared to norovirus-negative diarrheal illnesses, with pit latrines with slab bases the most common toilet facility among children with norovirus-positive episodes. Otherwise, there were no significant differences in the demographic or clinical features of norovirus-positive versus norovirus-negative diarrheal illnesses in those with available data in our cohort, including rotavirus vaccination status, duration of diarrhea, duration of fever, presence of blood in stool, vomiting, abdominal pain, fever, or rotavirus stool positivity (Table 1). Similarly, there were no significant differences in the clinical features of norovirus infections compared to rotavirus infections (data not shown). Similarly, when the severity of illness was compared according to the detection of norovirus or rotavirus, no significant differences were observed (norovirus-positive/rotavirus negative: 10/13 (77%) mild, 3/13 (23%) moderate; rotavirus-positive/norovirus negative: 66/84 (79%) mild, 18/84 (21%) moderate, norovirus-positive/rotavirus-positive 6/6 (100%) mild; *p* = 0.065). Similarly, no differences were observed in illness severity among GI compared to GII norovirus infections (Table 2).

**Table 2:** Severity score by norovirus genogroup* in young Zambian children with norovirus-associated diarrheal illness

	Genogroup I (*n* = 4)	Genogroup II (*n* = 10)	*p*-value
Severity score			1.000
< =10 (mild)	3 (75.0)	8 (80.0)	
11–14 (moderate)	1 (25.0)	2 (20.0)	
> =15 (severe)	0 (0.0)	0 (0.0)	

*Includes data only for subjects in whom severity score was calculable (*n* = 19) and genogroup was available (*n* = 14; one subject excluded due to co-infection with GI and GII)

## Discussion

We present the first report to our knowledge of the rate of norovirus infections among young children with diarrheal illness in Zambia using data obtained from a systematic surveillance study. The period prevalence of norovirus in these facilities was 11.5%, compared to 41% rotavirus period prevalence in this partially vaccinated population [7]. Diarrheal illness due to NoV is common among young children in this setting, and may become more important as rotavirus vaccine uptake increases. The clinical characteristics of norovirus positive and negative illnesses were not distinguishable. Several distinct norovirus genotypes were identified in this population, with GII represented in nearly three-quarters of the norovirus detections.

The recent finding that NoV replaced rotavirus as the leading etiology of childhood diarrhea after widespread rotavirus vaccine implementation in the United States [4] has generated substantial interest in defining the burden of norovirus disease in other regions. Several studies have examined the prevalence of norovirus detection among children presenting with diarrheal infections in sub-Saharan Africa. In the multicenter Global Enteric Multicenter Study (GEMS), conducted from 2007 to 2011, norovirus did not contribute substantially to mortality in children <5 years presenting to one of seven field sites in Asia or Africa with moderate-to-severe diarrhea [16]. In contrast, a multi-site birth cohort study of children utilizing prospective community surveillance conducted at several sites in South America, Asia, and Africa from 2009 to 2012 (Etiology, Risk Factors, and Interactions of Enteric Infections and Malnutrition and the Consequences for Child Health and Development Project; MAL-ED), found that NoV GII infections were associated with the highest or near-highest attributable burden of diarrheal illness in the first and second years of life [17]. Further, they found that 89% of children experienced at least one norovirus infection before 24 months, with similar disease severity to other enteropathogens [18]. The difference in these findings may be related to study design; while the GEMS study was designed as a case–control study and primarily recruited from health centers, the prospective birth cohort MAL-ED study captured household disease. Recent studies evaluating the prevalence of diarrheal pathogens among children with diarrhea in sub-Saharan Africa have reported NoV prevalence ranging from 9 to 25% among subjects with diarrhea, with typically 75 to 95% of symptomatic NoV detections associated with detection of NoV G.II [18–28]. In the studies that also enrolled asymptomatic controls, the prevalence of NoV in stool specimens obtained from asymptomatic subjects ranged from 3.9 to 31% and sometimes exceeded the prevalence in symptomatic subjects. However, cycle threshold values as surrogates for viral loads were useful in identifying clinically relevant NoV detections [21, 22]. Additionally, inter-study comparisons are limited by the use of different NoV detection methods as well as different definitions of cases and controls, reducing the generalizability of the two studies to other settings.

Our study has several important limitations. First, our surveillance was impaired by uneven collection of data. Clinical data were collected but were not systematically recorded in the first five months of the study, representing 37% of the total stool samples that were tested for norovirus. Study data collection forms were changed in November 2012; thus, the clinical features of illnesses prior to this transition are not captured. Additionally, stool samples were not collected from a substantial proportion of enrolled subjects (521/2027; 26%). The sample size of our study and the large number of missing stool samples are both important limitations, which may impact the generalizability of our results. In addition, our use of a convenience sample, rather than a random sample, may have introduced selection bias, as well as over- or under-representation of some months. Including both clinical and health care utilization parameters as enrollment criteria, some of which were subjective, may have resulted in misclassification bias. Additionally, we did not obtain samples from healthy control children without diarrhea for norovirus testing to inform the clinical significance of our results. Finally, the use of more restrictive primers may have underestimated the overall burden of norovirus in diarrheal illness in this population, and more sensitive, multiplex PCR tests may be applied to these samples in the future.

A significant difference identified in our study between norovirus-positive and norovirus-negative illnesses was latrine type. As oral-fecal contamination is an important route of transmission for norovirus and other enteric viruses, sanitation that allows appropriate disposal of feces may be an important intervention. However, given the wide variation of latrines prevailing in developing countries, it remains to be demonstrated whether improved latrines, such as those connected to a sewer line or with a lined sub-structure, may be associated with lower risk of infections such as norovirus.

## Conclusions

Norovirus was detected frequently from children < 5 years of age presenting with diarrhea in our sample derived from a health facility-based surveillance study. Longitudinal data will be needed to study changes in the relative importance of norovirus as a cause of clinically important diarrhea after widespread rotavirus vaccination implementation. Our findings and others may inform future strategies to prevent the morbidity and mortality associated with pediatric diarrheal infections.

## Additional file

Additional file 1: Figure S1. Phylogenetic analysis of a 280-bp region of the partial RNA dependent-RNA polymerase region in norovirus detections (1A: Genogroup I; 1B: Genogroup II). (PDF 79 kb)

## Abbreviations

AGE: Acute gastroenteritis
Ark: Absolute Return for Kids
CIDRZ: Centre for Infectious Disease Research in Zambia
EIA: Enzyme immunoassay
EPI: Expanded Programme on Immunisation
GEMS: Global Enteric Multicenter Study
GI and GII: Genogroup I and II
IPD: Inpatient department
MAL-ED: Etiology, Risk Factors, and Interactions of Enteric Infections and Malnutrition and the Consequences for Child Health and Development Project
NoV: Norovirus
OPD: Outpatient department
RT-PCR: Reverse transcriptase polymerase chain reaction

## References

[1] Koo HL, Ajami N, Atmar RL, DuPont HL (2010). Noroviruses: the leading cause of gastroenteritis worldwide. Discov Med.

[2] Ramani S, Atmar RL, Estes MK (2014). Epidemiology of human noroviruses and updates on vaccine development. Curr Opin Gastroenterol.

[3] Ahmed SM, Hall AJ, Robinson AE, Verhoef L, Premkumar P, Parashar UD, Koopmans M, Lopman BA (2014). Global prevalence of norovirus in cases of gastroenteritis: a systematic review and meta-analysis. Lancet Infect Dis.

[4] Payne DC, Vinje J, Szilagyi PG, Edwards KM, Staat MA, Weinberg GA, Hall CB, Chappell J, Bernstein DI, Curns AT (2013). Norovirus and medically attended gastroenteritis in U.S. children. N Engl J Med.

[5] Koo HL, Neill FH, Estes MK, Munoz FM, Cameron A, DuPont HL, Atmar RL (2013). Noroviruses: the most common pediatric viral enteric pathogen at a large university hospital after introduction of rotavirus vaccination. J Pediatr Infect Dis Soc.

[6] Chilengi R, Rudd C, Bolton C, Guffey B, Masumbu PK, Stringer J (2015). Successes, challenges, and lessons learned in accelerating introduction of rotavirus immunisation in Zambia. World J Vaccines.

[7] Beres LK, Tate JE, Njobvu L, Chibwe B, Rudd C, Guffey MB, Stringer JS, Parashar UD, Chilengi R (2016). A preliminary assessment of rotavirus vaccine effectiveness in Zambia. Clin Infect Dis.

[8] Beres LK, Tate JE, Njobvu L, Chilbwe B, Rudd C, Guffey M. Brad, Stringer, JAS, Parashar UD, Chilengi R. A preliminary assessment of rotavirus vaccine effectiveness in Zambia. Clin Infect Dis. 2016;62 Suppl 2:S175-82. 10.1093/cid/civ1206 PMC11976676 27059353

[9] Vinje J, Koopmans MP (1996). Molecular detection and epidemiology of small round-structured viruses in outbreaks of gastroenteritis in the Netherlands. J Infect Dis.

[10] Saitou N, Nei M (1987). The neighbor-joining method: a new method for reconstructing phylogenetic trees. Mol Biol Evol.

[11] Tamura K, Peterson D, Peterson N, Stecher G, Nei M, Kumar S (2011). MEGA5: molecular evolutionary genetics analysis using maximum likelihood, evolutionary distance, and maximum parsimony methods. Mol Biol Evol.

[12] Zharkikh A, Li WH (1995). Estimation of confidence in phylogeny: the complete-and-partial bootstrap technique. Mol Phylogenet Evol.

[13] Huhti L, Szakal ED, Puustinen L, Salminen M, Huhtala H, Valve O, Blazevic V, Vesikari T (2011). Norovirus GII-4 causes a more severe gastroenteritis than other noroviruses in young children. J Infect Dis.

[14] Ruuska T, Vesikari T (1990). Rotavirus disease in Finnish children: use of numerical scores for clinical severity of diarrhoeal episodes. Scand J Infect Dis.

[15] Wikswo ME, Desai R, Edwards KM, Staat MA, Szilagyi PG, Weinberg GA, Curns AT, Lopman B, Vinje J, Parashar UD (2013). Clinical profile of children with norovirus disease in rotavirus vaccine era. Emerg Infect Dis.

[16] Kotloff KL, Nataro JP, Blackwelder WC, Nasrin D, Farag TH, Panchalingam S, Wu Y, Sow SO, Sur D, Breiman RF (2013). Burden and aetiology of diarrhoeal disease in infants and young children in developing countries (the Global Enteric Multicenter Study, GEMS): a prospective, case–control study. Lancet.

[17] Platts-Mills JA, Babji S, Bodhidatta L, Gratz J, Haque R, Havt A, McCormick BJ, McGrath M, Olortegui MP, Samie A (2015). Pathogen-specific burdens of community diarrhoea in developing countries: a multisite birth cohort study (MAL-ED). Lancet Glob Health.

[18] Rouhani S, Penataro Yori P, Paredes Olortegui M, Siguas Salas M, Rengifo Trigoso D, Mondal D, Bodhidatta L, Platts-Mills J, Samie A, Kabir F (2016). Norovirus infection and acquired immunity in 8 countries: results from the MAL-ED study. Clin Infect Dis.

[19] Breurec S, Vanel N, Bata P, Chartier L, Farra A, Favennec L, Franck T, Giles-Vernick T, Gody JC, Luong Nguyen LB (2016). Etiology and epidemiology of diarrhea in hospitalized children from low income country: a matched case–control study in Central African Republic. PLoS Negl Trop Dis.

[20] Krumkamp R, Sarpong N, Schwarz NG, Adlkofer J, Loag W, Eibach D, Hagen RM, Adu-Sarkodie Y, Tannich E, May J (2015). Gastrointestinal infections and diarrheal disease in Ghanaian infants and children: an outpatient case–control study. PLoS Negl Trop Dis.

[21] Kabayiza JC, Andersson ME, Nilsson S, Bergstrom T, Muhirwa G, Lindh M (2014). Real-time PCR identification of agents causing diarrhea in Rwandan children less than 5 years of age. Pediatr Infect Dis J.

[22] Elfving K, Andersson M, Msellem MI, Welinder-Olsson C, Petzold M, Bjorkman A, Trollfors B, Martensson A, Lindh M (2014). Real-time PCR threshold cycle cutoffs help to identify agents causing acute childhood diarrhea in Zanzibar. J Clin Microbiol.

[23] Trainor E, Lopman B, Iturriza-Gomara M, Dove W, Ngwira B, Nakagomi O, Nakagomi T, Parashar U, Cunliffe N (2013). Detection and molecular characterisation of noroviruses in hospitalised children in Malawi, 1997–2007. J Med Virol.

[24] Oluwatoyin Japhet M, Adeyemi Adesina O, Famurewa O, Svensson L, Nordgren J (2012). Molecular epidemiology of rotavirus and norovirus in Ile-Ife, Nigeria: high prevalence of G12P[8] rotavirus strains and detection of a rare norovirus genotype. J Med Virol.

[25] Mans J, de Villiers JC, du Plessis NM, Avenant T, Taylor MB (2010). Emerging norovirus GII.4 2008 variant detected in hospitalised paediatric patients in South Africa. J Clin Virol.

[26] Mattison K, Sebunya TK, Shukla A, Noliwe LN, Bidawid S (2010). Molecular detection and characterization of noroviruses from children in Botswana. J Med Virol.

[27] Huynen P, Mauroy A, Martin C, Savadogo LG, Boreux R, Thiry E, Melin P, De Mol P (2013). Molecular epidemiology of norovirus infections in symptomatic and asymptomatic children from Bobo Dioulasso, Burkina Faso. J Clin Virol.

[28] Mans J, Murray TY, Nadan S, Netshikweta R, Page NA, Taylor MB (2016). Norovirus diversity in children with gastroenteritis in South Africa from 2009 to 2013: GII.4 variants and recombinant strains predominate. Epidemiol Infect.

