# Supplementary material for: Norovirus infections in young children in Lusaka Province, Zambia: clinical characteristics and molecular epidemiology
Source: BMC Infect Dis. 2017 Jan 23;17:92. doi: 10.1186/s12879-017-2206-2 (PMC5260028; doi:10.1186/s12879-017-2206-2)
Supplement: Additional file 1: Figure S1. — Phylogenetic analysis of a 280-bp region of the partial RNA dependent-RNA polymerase region in norovirus detections (1A: Genogroup I; 1B: Genogroup II). (PDF 79 kb) [file 12879_2017_2206_MOESM1_ESM.pdf]

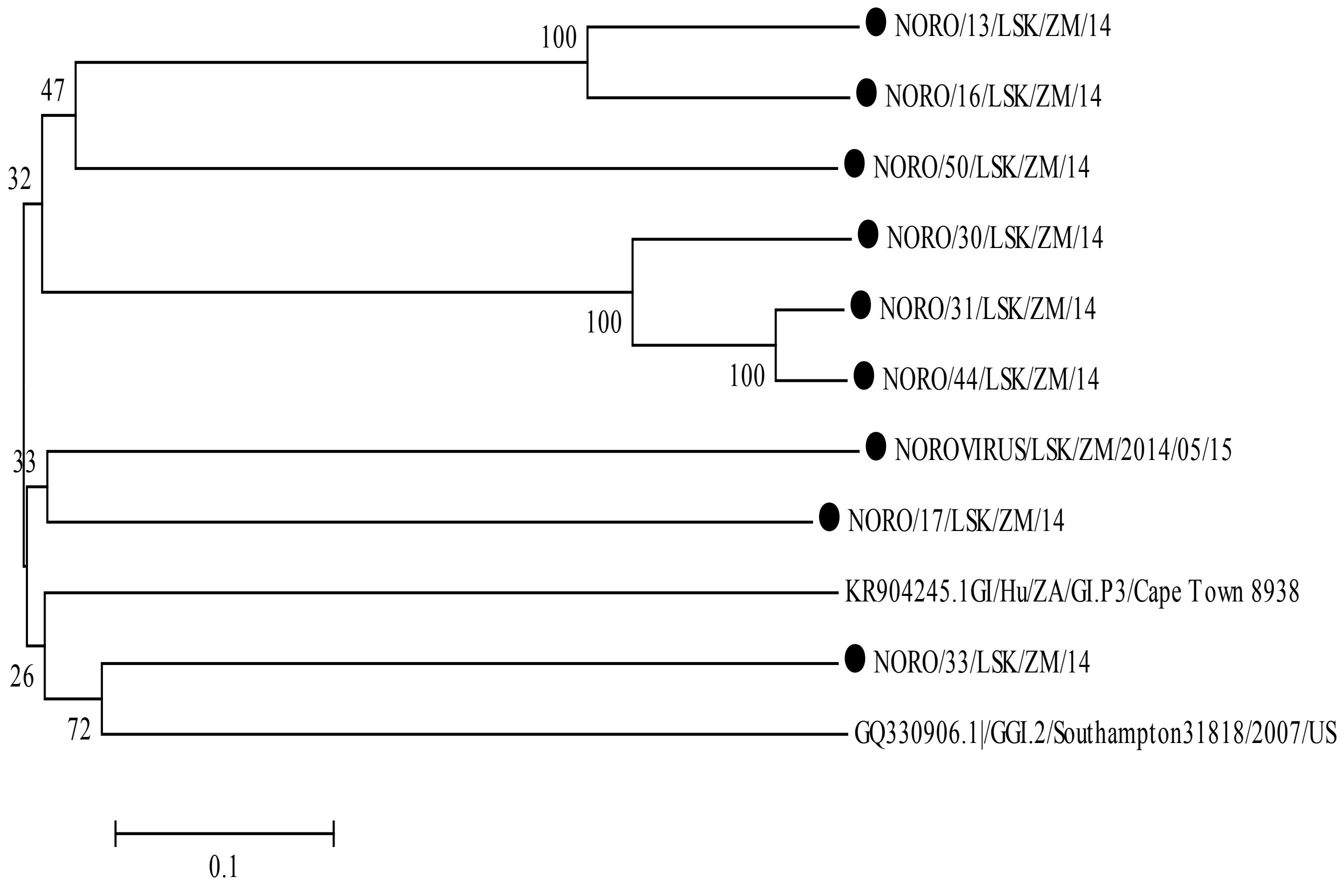

Supplemental Figure 1A. **Phylogenetic analysis of a 280-bp region of the partial RNA dependent-RNA polymerase region in NoV Genogroup I detections.** Nine genotypes of Genogroup I had sufficient length to be supported in the Mega software. Black circles represent sequences from our study compared to reference sequences from GenBank. NORO=norovirus/ Number of Isolation/Place of isolation/year of isolation; LSK=Lusaka. Branch lengths are indicative of the proportional to the evolutionary distance between sequences, and the distance scale in nucleotide substitutions per position is shown.

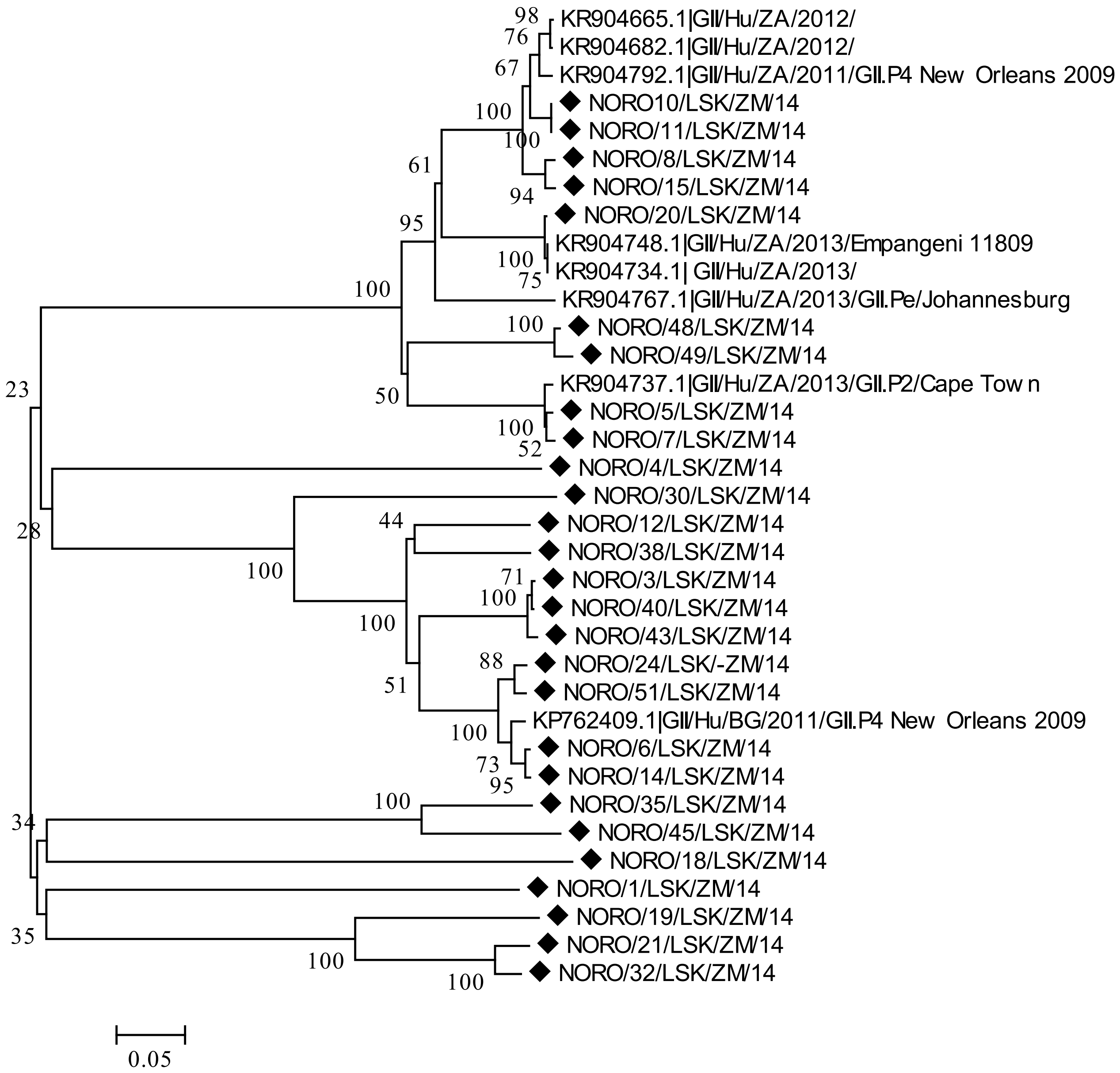

Supplemental Figure 1B. **Phylogenetic analysis of a 280-bp region of the partial RNA dependent-RNA polymerase region in NoV Genogroup II detections.** Twenty-seven genotypes of Genogroup II had sufficient length to be supported in the Mega software. Black circles represent sequences from our study compared to reference sequences from GenBank. NORO=norovirus/ Number of Isolation/Place of isolation/year of isolation; LSK=Lusaka. Branch lengths are indicative of the proportional to the evolutionary distance between sequences, and the distance scale in nucleotide substitutions per position is shown.
